# A new tale of lost tails: Correlates of tail breakage in the worm lizard *Amphisbaena vermicularis*

**DOI:** 10.1002/ece3.7023

**Published:** 2020-11-19

**Authors:** Jhonny J. M. Guedes, Henrique C. Costa, Mario R. Moura

**Affiliations:** ^1^ Programa de Pós‐Graduação em Ecologia e Evolução Departamento de Ecologia Universidade Federal de Goiás Goiânia Goiás Brazil; ^2^ Programa de Pós‐Graduação em Zoologia Departamento de Zoologia Universidade Federal de Minas Gerais Belo Horizonte Minas Gerais Brazil; ^3^ Departamento de Biologia Animal Museu de Zoologia João Moojen Universidade Federal de Viçosa Viçosa Minas Gerais Brazil; ^4^ Departamento de Ciências Biológicas Universidade Federal da Paraíba Areia Paraiba Brazil; ^5^ Present address: Departamento de Zoologia Instituto de Ciências Biológicas Universidade Federal de Juiz de Fora Juiz de Fora Minas Gerais Brazil

**Keywords:** Amphisbaenidae, autotomy, defensive behavior, natural history, tail loss, urotomy

## Abstract

Predator–prey interactions are important evolutionary drivers of defensive behaviors, but they are usually difficult to record. This lack of data on natural history and ecological interactions of species can be overcome through museum specimens, at least for some reptiles. When facing aggressive interactions, reptile species may exhibit the defensive behavior of autotomy by losing the tail, which is also known as “urotomy”. The inspection of preserved specimens for scars of tail breakage can reveal possible ecological and biological correlates of urotomy. Herein, we investigated how the probability of urotomy in the worm lizard *Amphisbaena vermicularis* is affected by sex, body size, temperature, and precipitation. We found higher chances of urotomy for specimens with larger body size and from localities with warmer temperatures or lower precipitation. There was no difference in urotomy frequency between sexes. Older specimens likely faced – and survived – more predation attempts through their lifetime than smaller ones. Specimens from warmer regions might be more active both below‐ and aboveground, increasing the odds to encounter predators and hence urotomy. Probability of urotomy decreased with increased precipitation. Possibly, in places with heavier rainfall worm lizards come more frequently to the surface when galleries are filled with rainwater, remaining more exposed to efficient predators, which could result in less survival rates and fewer tailless specimens. This interesting defensive behavior is widespread in squamates, but yet little understood among amphisbaenians. The novel data presented here improve our understanding on the correlates of tail breakage and help us to interpret more tales of lost tails.

## Graphical abstract

Preserved specimens can be used to improve our understanding on the behavioral ecology of reptiles. In inspecting scars of tail breakage in preserved specimens of worm lizards, the chances of tail loss increased with body size and temperature, and decreased with precipitation. Tail loss was not affected by sex, suggesting similar predation pressure on both male and female worm lizards.

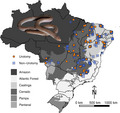

## 1 INTRODUCTION

Predator–prey interactions are important evolutionary drivers of defensive behaviors, with prey survivorship relating to either predator avoidance or antipredator behaviors (Brodie et al., 1991). Although antipredator mechanisms are known for several different animal groups (Edmunds, 1974), behavioral observations in nature might prove difficult. Most species are naturally rare (Preston, 1948), and in situ observations of predator–prey interactions are generally scarce (Fitch, 1987). However, for many species this difficulty can be overcome through the inspection of specimens housed in scientific collections. For instance, individuals from different animal groups, when attacked by predators or intraspecific competitors, may exhibit autotomy – self‐controlled behavior of losing a body part (Emberts et al., 2019), and ultimately survive (Arnold, 1984). If later collected and housed in a scientific collection, the preserved specimen can be used to retrieve information on autotomy and its potential determinants. This interesting behavior evolved independently multiple times across invertebrates and vertebrates, and a diverse set of appendages can be autotomized, with implications for predator–prey interactions, intraspecific competition, movement, and habitat selection (Emberts et al., 2019; Fleming et al., 2007).

Reptiles shed their tails during autotomy, and the term “urotomy” has been proposed as a refined terminology to describe all types of tail breakage in this group (Slowinski & Savage, 1995). Urotomy involves intra‐ or intervertebral tail breakage, followed or not by the regeneration of the tail. Briefly, autonomous intravertebral tail breakage is observed in the tuatara and many lizard groups, with posterior regeneration of the tail (Arnold, 1984; Bateman & Fleming, 2009; Etheridge, 1967). Conversely, intervertebral breakage is observed in some snakes, under passive fracturing of adjacent caudal vertebrae, without regeneration (Arnold, 1988; Costa et al., 2014; Crnobrnja‐Isailović et al., 2016; Slowinski & Savage, 1995). Intermediate conditions are observed in agamid lizards, with intervertebral tail breakage followed by regeneration (Arnold, 1988; Slowinski & Savage, 1995), and in some worm lizards (Amphisbaenia), that show intravertebral tail breakage without regeneration (Gans, 1978). Despite the growing number of reports on urotomy, especially for lizards and snakes (e.g., Bateman & Fleming, 2009; Crnobrnja‐Isailović et al., 2016), our knowledge on the factors affecting the urotomy probability in reptiles is still inadequate (Bustard, 1968; Kuo & Irschick, 2016; Smith, 1996), but particularly worse for worm lizards because of their fossorial lifestyle (Gans, 1978; Colli et al., 2016).

The family Amphisbaenidae comprises about 90% of all worm lizard species (Uetz, Freed, & Hosek, 2019), and urotomy occurs in most taxa, particularly – but not restricted to – smaller ones (Mott & Vieites, 2009). Tail breakage occurs only once (without regeneration) at a single fracture plane, usually externally visible as a narrowed, shortened, or differently pigmented ring of scales, known as “autotomy annulus” (Gans, 1978). Autotomy in Amphisbaenidae occurs at the proximal portion of the tail, mostly between the fifth and eighth caudal rings – tail range from about 13–40 rings (Vanzolini, 2002). Herein, we used preserved specimens of *Amphisbaena vermicularis* Wagler, 1824 as a model organism to retrieve information on urotomy and investigate its potential correlates. This species is widely distributed, occurring from northeastern Brazil to southeastern Bolivia, mostly along the South American “diagonal of open formations” (Gans & Amdur, 1966; Colli et al., 2016). It is a medium‐sized amphisbaenian (max. 370 mm) with its tail representing about 12% of its total body length (Gans & Amdur, 1966). Little is known about the natural history of *Amphisbaena vermicularis*. Specimens may eventually forage above the ground (Aragão et al., 2019), and there are predation records by snakes (França et al., 2008; Lisboa & Freire, 2010; Oliveira et al., 2014), a frog (Vaz‐Silva, Silva, & Silva Junior, 2003), and birds (Nolasco et al., 2020). Aggressive intraspecific interactions or even defensive behaviors are not reported for *A. vermicularis*, except for observations of captive specimens “jumping” to escape when placed over hard soil – unable to dig as an escaping mechanism (Navega‐Gonçalves & Benites, 2019). However, as reported for other South American species * * (Brito et al., 2001), tail autotomy may indeed represent an antipredatory behavior for *A. vermicularis*. We aimed to assess four hypotheses regarding the correlates of urotomy in *A. vermicularis*:

1. Although male and female squamates can differ in body size and behavior, most studies on urotomy have not found evidence of sexual differences in the frequency of tail breakage in lizards (Bateman & Fleming, 2009) and snakes (see Costa et al., 2014 and references therein), as well as for amphisbaenians (Papenfuss, 1982). Moreover, *Amphisbaena vermicularis* does not show sexual size dimorphism in body length (H.C. Costa, pers. obs.); thus, we expect a lack of sex effect on the occurrence of urotomy for this species.
2. A positive relationship between frequency of urotomy and body size has been reported for many reptiles (Papenfuss, 1982; Willis et al., 1982; Vitt & Cooper, 1986; Costa et al., 2014). This possibly reflects a longer “exposure time” of older specimens to predators compared with younger ones (Vitt & Cooper, 1986). Alternatively, this could be attributed to morphological and behavioral differences between specimens of distinct body size (Daniels et al., 1986; Vitt & Cooper, 1986). We expect higher incidence of tail breakage in large relative to small specimens of *A. vermicularis*.
3. Reptiles are ectothermic animals, and their ecology is highly dependent on the regulation of body temperature (Pianka & Vitt, 2003). We expect specimens from warmer localities to be more active, both below‐ and aboveground, which could potentially increase their chances of being found by predators, and therefore, of showing urotomy scars as well.
4. During heavy rainfalls, underground galleries are filled with water and worm lizards are forced to emerge to the surface (Bates, 1993). We expect that specimens from localities with heavier rainfalls will present higher frequency of urotomy because they are more exposed to predators above the ground and therefore likely exhibit high frequency of urotomy.

If we assume that inter‐ and intraspecific competition is negligible for tail loss in *Amphisbaena vermicularis*, urotomy frequency can be interpreted as an indicative of predation intensity or efficiency. Despite criticism regarding the use of urotomy frequency as an index of predation (Jaksić & Busack, 1984; Jaksić & Greene, 1984), this seems a valid assumption for *A. vermicularis* since there is no evidence suggesting that intraspecific competition plays any role in tail breakage of worm lizards; the lost tail keeps moving after being autotomized, which could distract predators (Navega‐Gonçalves & Benites, 2019; Papenfuss, 1982); and the tail can only be shed once (Gans, 1978), making it a unique antipredatory behavior (Brito et al., 2001). Regardless of these issues, we herein hope to reduce existing knowledge gaps between worm lizards and other squamates regarding patterns and processes of urotomy.

## 2 MATERIALS AND METHODS

### 2.1 Data collection

**Figure 1:**
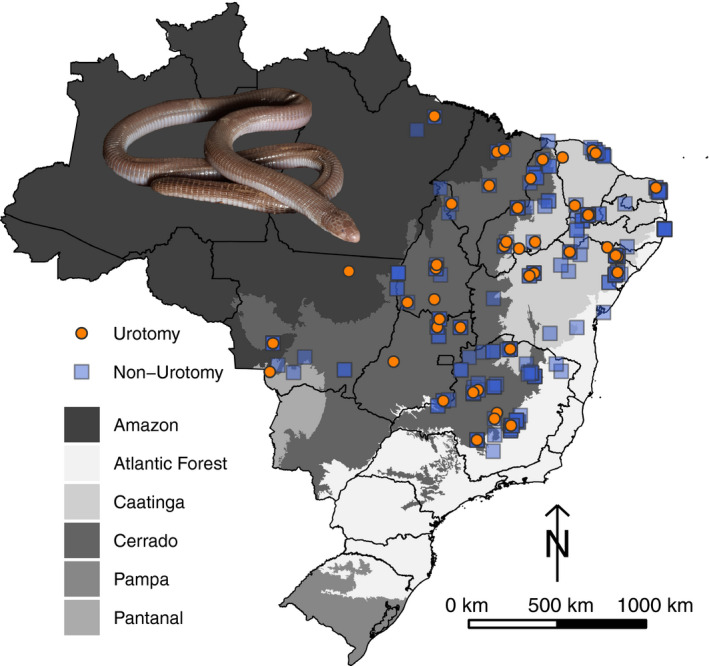
Geographic distribution of specimens of *Amphisbaena vermicularis* analyzed in this study. Photograph of the specimen by R. Gaiga

We examined a total of 396 preserved specimens of *Amphisbaena vermicularis*, housed in 22 scientific collections (see raw data in the Data Availability section). Specimens were collected along most of the species’ geographic range, covering all ecoregions from where it is known to occur, except by the Chiquitano dry forests and the Dry Chaco of Bolivia (Figure 1). For each specimen, we recorded the condition of the tail tip as a binary variable (healed broken tail = urotomy =1; intact rounded tail = no urotomy = 0). Urotomy was not considered for unhealed broken tails because it was not possible to confirm whether breakage occurred before or after collection, since the tail can break during the handling of preserved specimens. Overall, we removed 50 specimens from our dataset due to uncertainty in the presence of urotomy, resulting in 346 specimens.

In addition, potential differences in survival between life stages could affect urotomy probability. Juveniles may be less mobile and use habitats that provide more cover to predators or, conversely, have poor escaping abilities than adults, ultimately leading to different survival rates (Pike et al., 2008; Kacoliris, Berkunsky, & Velasco, 2013). We obtained the life stage of each specimen based on the known size at sexual maturity for males (176 mm SVL; Santos, 2013) and females (193 mm SVL; H.C. Costa, pers. obs.) of *Amphisbaena vermicularis* – sexing procedures are defined below. In the case of doubtful sex identification, we used the threshold of 193 mm SVL to consider unsexed specimens as adults. Our dataset had a total of 303 adults, 39 juveniles, and 4 specimens which life‐stage identification was not possible due to poor preservation condition. Because of the low sample size for juveniles, we modeled urotomy probability based only on adult specimens.

We recorded four covariates for each specimen: (a) sex – determined based on the presence or absence of the hemipenes through a longitudinal incision at the base of tail, or by checking the gonads through a small longitudinal incision at the belly; (b) body size – represented by the snout–vent length and measured with a ruler to the nearest 1 mm from the tip of the snout to the posterior border of the pre‐cloacal plates; and the (c) mean annual temperature and (d) precipitation in the wettest quarter at the collection site of each specimen. The extraction of the latter two covariates was based on the geographic coordinates of each specimen as informed in the specimens catalogue (available in scientific collections), gazetteers (IBGE, 2011; Paynter & Melvin, 1991; Vanzolini, 1992), or manually obtained via Google Earth Pro. We assumed precipitation in the wettest quarter to better represent the chances of experiencing heavier rainfalls in a region. Both environmental variables were extracted from the WorldClim database at the spatial resolution of 5 arc‐min (Fick & Hijmans, 2017). We excluded five out of 303 specimens because of missing data for body size (poor preservation condition) or geographic coordinates, thus resulting in a final dataset with 298 adult specimens.

### 2.2 Statistical analysis

Since our response variable (urotomy) is binary, we analyzed the data through a logistic regression – that is, a generalized linear model (GLM) with a binomial error distribution. We used four predictors, three of which are continuous (body size, temperature, precipitation) and one categorical (sex). Our aim was to investigate how those environmental and intrinsic biological predictors affected the probability of urotomy in *A. vermicularis*. We verified the skewness and kurtosis of the continuous variables, but none needed to be transformed. We also checked for multicollinearity among predictors using variation inflation factors (VIF; Mansfield & Helms, 1982). A predictor holding VIF higher than 10 indicate strong multicollinearity, and means it should be removed from analysis (Kutner et al., 2005), but none of our predictors reached values higher than two.

We initially tested the effect of sex on urotomy frequency separately due to the need to reduce our sample size by removing 51 adult specimens with doubtful sex identification (there were 252 sexed specimens). However, since preliminary analyses showed no sexual difference in urotomy frequency (chi‐square test: χ*^2^* = 0.193, *df* = 1, *p* = .659), we pooled all adult specimens to avoid reducing sample size and statistical power and proceeded without the sex covariate in further analysis. We used a backward variable selection procedure based on the likelihood ratio test (LRT) to keep in our model only significant predictors. Briefly, we started the variable selection procedure by computing LRTs between the full model (containing body size, temperature, and precipitation) and all possible models with the removal of one predictor. At each iteration, we registered the LRT value and the respective p‐value associated with a simplified model (i.e., dropping one predictor). A given predictor was removed from model if it scored both the lowest LRT and *p* > .05. The procedure was repeated until the selected model had only significant predictors (*p* ≤ .05).

### 2.3 Sensitivity analysis

Most specimens included in our analysis did not present urotomy (232 out of 298, or 77.9% of the total). Therefore, to account for the potential influence of unbalancing in the logistic regression, we performed a sensitivity analysis. We created data subsets with three different proportions of “urotomized” specimens (urotomy versus. non‐urotomy; 30:70, 40:60, 50:50). For all subsets, we kept all 66 observations of urotomy and randomly sampled the observations of non‐urotomy (without replacement) until achieving the desirable proportion between urotomized and non‐urotomized specimens. Overall, we created 10,000 data subsets for each proportion of urotomized specimens (i.e., 30, 40, or 50%), totaling 30,000 data subsets. For each data subset, we performed the variable selection procedure (see above) and stored the LRT value before dropping a given predictor from the model (if the predictor was selected to be removed). Then, for each proportion of urotomized specimens, we averaged the 10,000 LRT values computed for each predictor and calculated the respective p‐value according to the chi‐square distribution. Computations were performed in the software R version 3.3.3 (R Core Team, 2019). See Data Availability section for R scripts and raw data.

## 3 RESULTS

The overall frequency of tail breakage in *Amphisbaena* *vermicularis* was 19.9% across all specimens (69 out of 346 specimens) and 22.1% (66 out of 298 specimens) among the analyzed specimens (adults only). Of the 252 sexed adult specimens, urotomy was present in 30 males (20.8%, *n* = 144 sexed specimens) and 25 females (23.1%, *n* = 119). The frequency of urotomy did not differ between sexes in adult specimens (Chi‐square test: χ *^2^* = 0.19, *df* = 1, *p* = .659).

For the analysis performed with adult specimens regardless of sex (*n* = 298), all three predictors were significant. Body size presented the highest effect size (Std. coef. = 0.527, LRT: χ^2^ = 13.7, *p* < .001), positively affecting the probability of urotomy in *A. vermicularis*. As expected, specimens from warmer regions showed high probability of urotomy (Std. coef. = 0.386, LRT: χ^2^ = 5.669, *p* = .017), but contrary to our expectation, the probability of urotomy decreased among specimens from wetter regions (Std. coef. = −0.322, LRT: χ^2^ = 4.80, *p* = .028).

**Figure 2:**
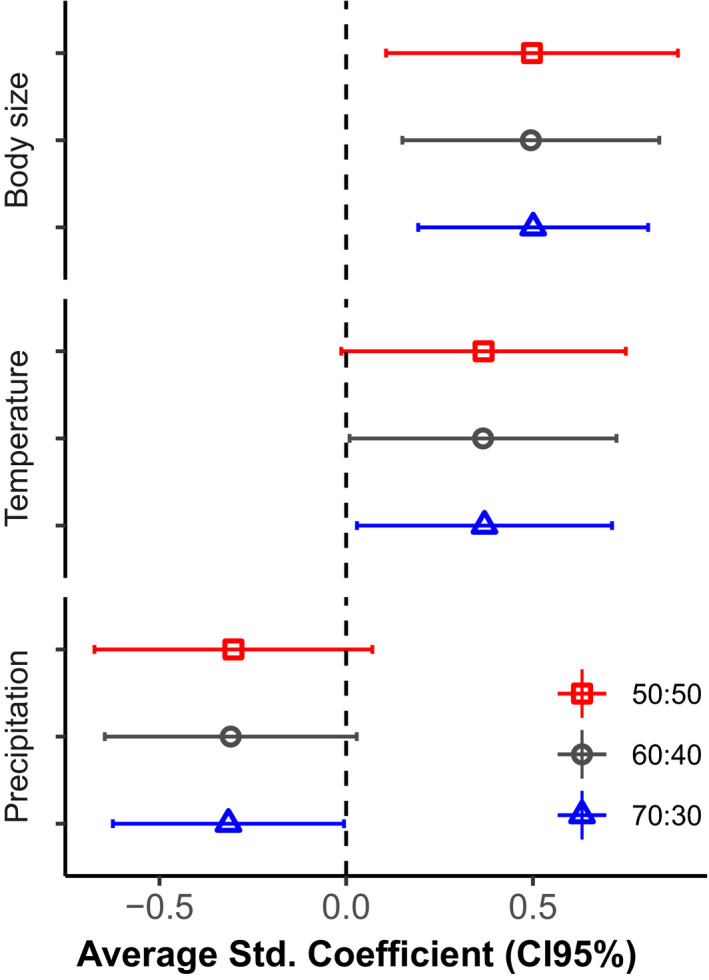
Sensitivity analysis and its averaged standardized coefficients from the logistic regression model for each proportion of unbalancing between non‐urotomized and urotomized specimens of *Amphisbaena vermicularis*. The horizontal bar denotes 95% confidence intervals.

After repeating the analysis with controlled levels of unbalancing in our response variable (urotomized versus. non‐urotomized specimens), we detected some differences among the selected predictors. Body size was the only predictor showing consistent relationship with probability of urotomy across all levels of unbalance in the response variable (average LRT: χ^2^ = 10.94, *p* < .001 for 30:70 [*n* = 218]; χ^2^ = 8.86, *p* = .002 for 40:60 [*n* = 165]; χ^2^ = 7.19, *p* = .007 for 50:50 [*n* = 132]; Figure 2). We detect an effect of temperature in two levels of unbalance (average LRT: χ^2^ = 5.06, *p* = .024 for 30:70; χ^2^ = 4.58, *p* = .032 for 40:60), and precipitation was important only in the proportion of 30:70 (average LRT: χ^2^ = 4.13, *p* = .042) (Figure 2).

## 4 DISCUSSION

Tail autotomy is an interesting but yet understudied defensive behavior among amphisbaenians, especially regarding its underlying mechanisms. We have shown the role of biological and environmental correlates on the probability of urotomy in *Amphisbaena vermicularis* in Brazil. About 23% of preserved specimens of this species show healed broken tails, but without differences in frequency between adult males and females. The probability of urotomy increased with body size and temperature, but decreased with precipitation. The role of environmental correlates was reduced after controlling for the unbalancing in the proportion of urotomized and nonurotomized specimens (Figure 2).

Frequencies of urotomy in squamates vary among species or even intraspecifically (Arnold, 1984). For example, sexual differences may arise because of behavioral differences between males and females (Papenfuss, 1982). Despite the lack of empirical evidence of male–male competition in amphisbaenians, experiments show that males respond aggressively to scents of conspecific males (López & Martín, 2009; Martín et al., 2020). Thus, sexually dimorphic species where males are larger than females (e.g., Gomes et al. 2009; Souza e Lima et al. 2014) could indicate male–male competition, ultimately leading to tail breakage. There is no sexual dimorphism in *Amphisbaena vermicularis*, and we found no sexual differences in the frequency of urotomy between sexes, which suggests that tail loss does not involve differential survival between males and females (Costa et al., 2014). The lack of sexual differences in the frequency of urotomy seems to be a common pattern for many squamates, including some species with obvious morphological and behavioral differences between sexes (Bateman & Fleming, 2009; Papenfuss, 1982).

The probability of urotomy increases with body size in *Amphisbaena vermicularis*, with larger specimens showing higher probability of urotomy than smaller ones. The positive relationship between body size and tail loss has been consistently found in other studies (e.g., Papenfuss, 1982; Willis et al., 1982; Vitt & Cooper, 1986; Costa et al., 2014; but see Jaksić and Fuentes, 1980). This may occur because older specimens are likely to face (and survive) more predation attempts than younger and smaller ones during their lifetimes (Daniels et al., 1986; Mendelson, 1992; Papenfuss, 1982). However, tail breakage could have occurred in juveniles that survived and reached adulthood. Indeed, survival rates of juvenile reptiles seem higher than previously thought (Pike et al., 2008), making this possibility not so unlikely. Therefore, the temporal aspect of urotomy cannot be established for certain without, for example, long‐term capture–mark–recapture experiments (e.g., Kuo & Irschick, 2016).

Variation in environmental conditions is known to affect the probability of urotomy in different ways. For instance, the common side‐blotched lizard, *Uta stansburiana*, and the gecko *Phyllodactylus marmoratus*, lose their tail more easily at higher environmental temperatures (Brattstrom, 1965; Daniels, 1984), while in the gecko *Gehyra variegata,* urotomy is bimodal – tails are readily lost at extreme temperatures (Bustard, 1968). Specimens of *Amphisbaena vermicularis* from warmer regions show, to some extent, high probability of urotomy relative to those experiencing colder temperatures. Amphisbaenians are ectothermic and rely on external sources to warm their bodies, which is usually done by basking under rocks, or moving through thermal gradients within the soil (Balestrin & Cappellari, 2011; López, Civantos, & Martín, 2002; López, Salvador, & Martín, 1998; Matias & Verrastro, 2018; Papenfuss, 1982). The lack of data on thermal ecology and activity patterns of *A. vermicularis* prevents robust conclusions on the role of temperature in probability of urotomy, but at least three factors are possible. Firstly, *A. vermicularis* from warmer localities spend more time buried at greater depths to avoid overheating, being more susceptible to either intraspecific aggressions or predation attempts by fossorial predators like some snakes (e.g., *Apostolepis*, *Micrurus*, and *Phalotris*), thus making urotomy more frequent. Secondly, it is possible that *A. vermicularis* shows a high voluntary thermal maximum – that is, maximum environmental temperature that an animal supports before seeking shelter – like *A. alba* (Díaz‐Ricaurte & Serrano, 2020), and individuals from warmer localities spend more time active on the surface (Aragão et al., 2019) or the subsurface, increasing their susceptibility to aboveground predators (e.g., birds and mammals), also resulting in more urotomy. Lastly, higher temperatures may result in greater thermal efficiency and increase escaping rates of *A. vermicularis*, so worm lizards simply survive more frequently to predation (by losing the tail) than those from areas with lower temperatures.

During heavy rainfalls, underground galleries are filled with water and worm lizards might be forced to surface (Bates, 1993), which could make them more exposed to aboveground predators (birds and mammals; e.g., Hayes et al., 2016) and increase the frequency of urotomy. Contrary to our expectations, the probability of urotomy in *Amphisbaena vermicularis* may decrease with precipitation. Possibly, in places with higher precipitation (and heavy rainfalls), amphisbaenians indeed come to the surface more frequently to avoid drowning, but fewer specimens may end up surviving opportunistic predation because of, for example, higher predation intensity or efficiency (Bateman & Fleming, 2009). Alternatively, *A. vermicularis* might show less fossoriality than initially thought and its survival be affected by refuge availability. The scarce data available on the diet of *A. vermicularis* inform individuals foraging either on the surface (Aragão et al., 2019) or having surface‐dwelling ants as prey (Esteves et al., 2008), which, in concern with the species countershading color pattern, suggest some level of surface activity (Gans, 1968). Thus, specimens foraging in regions with low precipitation may benefit from high refuge availability – that is, underground galleries not filled with water – and escape predators more often than specimens facing heavy rains.

Reports on frequency of tail breakage are scarce for amphisbaenians, but information available in the literature can provide some insights (Table 1). The available data show that urotomy frequency range from 2.7% in the Mexican species *Bipes biporus* (Papenfuss, 1982) to 50% in the African *Loveridgea ionidesii* (Gans & Kraklau, 1989). Most often, urotomy frequencies lie below a 25% threshold, which is also the case for *Amphisbaena vermicularis* (Table 1). These frequencies are much lower than, for example, those commonly observed in snakes (see Costa et al., 2014 and references therein). Such low frequency of urotomy could result from at least two distinct processes: (a) low “predation intensity” (Pianka, 1970) – that is, few predators or low predator abundance may reduce the frequency of predation attempts; or (b) high “predation efficiency” (Schoener, 1979) – that is, prey are less efficient to escape their predators, and therefore, there are less specimens to “tell the tale”. The fossorial lifestyle of amphisbaenians reduces considerably their potential predators (see supplementary material in Schalk & Cove, 2018), which may decrease predation attempts and urotomy (Papenfuss, 1982), but the scarcity of natural history data for most amphisbaenians makes this still unclear. Most importantly, differently from most lizards that regenerate their broken tails and some snakes with the possibility of multiple tail breakage, amphisbaenians can lose their tail only once because they have a single fracture plane and regeneration does not occur (Arnold, 1984; Gans, 1978). The uniqueness of this defensive behavior in worm lizards could decrease the probability of encountering specimens with urotomized tails since this defensive mechanism can only be used once.

**Table 1:** Frequency of urotomy for amphisbaenian species available in the literature. We included only those species that frequency is based on at least 50 specimens

Species	Specimens analyzed (*n*)	Urotomy frequency (%)	References
*Bipes biporus* ^a^	–	2.7	Papenfuss (1982)
*Amphisbaena kingii*	73	4.1	Gans & Rhodes (1964)
*Bipes canaliculatus* ^a^	–	10.2	Papenfuss (1982)
*Amphisbaena darwinii*	443	15.6	Gans (1966b)
*Chirindia swynnertoni* ^b^	90	16.5	Broadley and Gans (1978b)
*Bipes tridactylus* ^a^	–	17.0	Papenfuss (1982)
*Chirindia langi* ^b^	58	18.5	Broadley and Gans (1978b)
*Amphisbaena vermicularis*	346	19.9	This study
*Amphisbaena fuliginosa*	129	21.7	Vanzolini (1951)
*Amphisbaena vermicularis*	144	22.9	Gans and Amdur (1966)
*Zygaspis violacea* ^b^	104	24.0	Broadley and Gans (1978a)
*Amphisbaena mertensii*	78	32.1	Gans (1966a)
*Loveridgea ionidesii*	459	50.0	Gans and Kraklau (1989)
*Monopeltis guentheri*	89	15–20	Gans and Latifi (1971)

^a^ The author did not specify the number of specimens, but more than 3,800 specimens of the three *Bipes* species were collected during field expeditions for that study.

^b^ Number of specimens obtained from the material examined.

We recognize that controlled laboratory and field experiments are needed for a clear distinction of factors affecting biotic interactions and indirectly causing autotomy among different taxa and geographic locations (e.g., Itescu et al. 2016). For instance, the use of caterpillar clay models indicated higher predation at lower latitudes and elevations for arthropod predators, but not for bird and mammal predators (Roslin et al., 2017). Although mammals and birds predate upon worm lizards, fossorial snakes are among the most common predators reported in the literature (Schalk & Cove, 2018). Probably, fossorial snake predation upon worm lizards is even higher due to difficult of detecting predation events below ground. Encounters between fossorial snakes and worm lizards potentially occur underground, in a face‐to‐face or face‐to‐tail manner, which might improve the odds of tail autotomy relative to encounters with bird and mammal predators. Considering that predation intensity by endotherms might not change latitudinally (Roslin et al., 2017), and snake movement and activity increase with temperature (Eskew & Todd, 2017), it is possible that higher autotomy rates in worm lizards from warmer regions reflect high predation intensity by fossorial snakes. Further investigations are needed to shed light on the mechanisms by which biological and environmental factors affect autotomy rates.

Because of their fossorial lifestyle, worm lizards are underrepresented in most scientific collections and figure among the poorest known vertebrates groups (Colli et al., 2016). However, for some species an adequate number of preserved specimens allow the investigation of many ecological processes and its determinants, as shown here for the correlates of tail autotomy in a tropical worm lizard species. While field experiments are still insufficient and proper resources largely unavailable, the use of preserved specimens may be the only source of ecological information at hand to advance our knowledge on the ecology of most tropical species.

## CONFLICT OF INTEREST

Authors declare they have no conflict of interest.

## AUTHOR CONTRIBUTION

Jhonny José Magalhães Guedes: Data curation (equal); Formal analysis (equal); Methodology (equal); Writing‐original draft (equal). Henrique C. Costa: Conceptualization (equal); Data curation (equal); Supervision (equal); Writing‐review & editing (equal). Mario R. Moura: Formal analysis (equal); Methodology (equal); Supervision (equal); Writing‐review & editing (equal).

## Data Availability

The R‐script and raw dataset supporting the results of this work are available at Dryad Digital Repository (https://doi.org/10.5061/dryad.pnvx0k6kd, Guedes et al., 2020).
